# Mr. Reid, in Reply to Mr. Potts

**Published:** 1801-12

**Authors:** J. R. Reid

**Affiliations:** Surgeon. Chelsea


### *To the Editors of the Medical and Phyfical Journal.*

GENTLEMEN,

IN the letter I addreffed to you in April laft, on the fubject of Mr. Pott's artificial leg, I flattered myfelf that I had been fo extremely guarded in my expreffions, as to have avoided every poffibility of giving that gentleman offence. I find, however, that neither the precaution I ufed, nor the advice Mr. Sheldrake gave, have been fufficient to prevent Mr. P. replying to me, with a degree of ill humour, which I can only attribute to chagrin and jealoufy, occafioned by feeing a rival invention patronized and recommended by fo many refpectable members of the profeffion.

Mr. Mann's patent leg was introduced to my notice, feveral years fince, by two gentlemen, who (uniting a high degree of mechanical knowledge with the information neceffarily derived from their fituations, as furgeons to two of the largeft hofpitals in the metropolis) might be confidered fully adequate to judge of the merits of an inftrument of this kind; they imagined my profeffion, and the appointment I held at Chelfea Hofpital, might afford me opportunities of giving it that publicity they thought it entitled to; and, from that period, I certainly have, "with the feelings of a faithful fteward," endeavoured to promote Mr. Mann's intereft, and I truft, in fome meafure alfo, the advantage of thofe whofe misfortune rendered this machine peculiarly interefting to them.

My affertion of Mr. Mann's title to the credit of having *firft* difcovered an inftrument, poffeffing certain properties, had immediate immediate reference to Mr. Potts's claim of priority on the ſame ſcore, a claim he is now obliged to abandon; and thus far my aſſertion will be found ſtrictly and literally true. In regard to productions of this kind, antecedent to Mr. Mann's, if there really were any, they certainly were not held in ſufficient eſtimation to make them objects of public notoriety; and in this point of view, I repeat my aſſertion, that Mr. M. was the *first person* who produced an artificial leg, poſſeſſing the qualities aſcribed to his, in ſuch a degree of perfection as to excite the attention and approbation of ſo many high profeſſional characters. If Mr. Potts's diſcovery "has merit enough to bear a compariſon with any other intended for the ſame purpoſe," let him patiently and quietly await that teſt, to which, I repeat, (though I may again expoſe myſelf to the charge of "an inſidious affectation of candour,") I wiſh their reſpective claims to be ſubmitted, *that of experience.* By doing this, and following Mr. Sheldrake's excellent advice, of "avoiding to engage in any public controverſy on the ſubject," he will render more ſervice to his cauſe, than by filling twenty pages with peeviſh and uſeleſs diſputation, (the certain evidence of mortification and diſappointment). At all events, gentlemen, I am determined to reap the benefit of his friend's admonition, and therefore promiſe you never again on this ſubject to occupy any part of a publication that would otherways be ſo much better employed. I am, &c.

*Chelsea, Nov.* 1801. J. R. REID, Surgeon.

